# SIRT1 Regulates Dendritic Development in Hippocampal Neurons

**DOI:** 10.1371/journal.pone.0047073

**Published:** 2012-10-04

**Authors:** Juan F. Codocedo, Claudio Allard, Juan A. Godoy, Lorena Varela-Nallar, Nibaldo C. Inestrosa

**Affiliations:** Departamento de Biología Celular y Molecular, Centro de Envejecimiento y Regeneración (CARE), Facultad de Ciencias Biológicas, P. Universidad Católica de Chile, Santiago, Chile; INSERM U894, France

## Abstract

Dendritic arborization is required for proper neuronal connectivity. SIRT1, a NAD+ dependent histone deacetylase, has been associated to ageing and longevity, which in neurons is linked to neuronal differentiation and neuroprotection. In the present study, the role of SIRT1 in dendritic development was evaluated in cultured hippocampal neurons which were transfected at 3 days *in vitro* with a construct coding for SIRT1 or for the dominant negative SIRT1H363Y, which lacks the catalytic activity. Neurons overexpressing SIRT1 showed an increased dendritic arborization, while neurons overexpressing SIRT1H363Y showed a reduction in dendritic arbor complexity. The effect of SIRT1 was mimicked by treatment with resveratrol, a well known activator of SIRT1, which has no effect in neurons overexpressing SIRT1H363Y indicating that the effect of resveratrol was specifically mediated by SIRT1. Moreover, hippocampal neurons overexpressing SIRT1 were resistant to dendritic dystrophy induced by Aβ aggregates, an effect that was dependent on the deacetylase activity of SIRT1. Our findings indicate that SIRT1 plays a role in the development and maintenance of dendritic branching in hippocampal neurons, and suggest that these effects are mediated by the ROCK signaling pathway.

## Introduction

Neurons are highly polarized cells, most of which have a main axon that relays information to other neurons and dendrites that typically receive inputs from other neurons. Early neuronal development consists of a stereotypic progression of events beginning with neurite extension, differentiation of axon, dendritic arborization and synaptic formation [1]. Dendritic arborization correlates with the number and distribution of inputs that each neuron can receive and process.

SIRT1 has been linked as a major player in caloric restriction response and life span control, SIRT1 is localized mainly to the nucleus and is functionally linked to insulin growth factor (IGF) signaling pathway and activation of FOXO transcription factors, NF-κβ, p53, and induces the expression of their target genes involved in stress protection, while inhibiting other target genes involved in cell cycle arrest, senescence or apoptosis [2], [3]. During neuronal differentiation SIRT1 is involved in the redox potential sensing, and this redox potential determines whether SIRT1 drives cell towards neuronal or astroglial cell fates [4]. Under normal conditions SIRT1 is important for neuronal differentiation, and its nuclear activity is critical for its function [5].

The induction and activation of SIRT1 by caloric restriction in the brain has been linked to the integrity of neurons. SIRT1 has a neuroprotective role against Parkinson and Huntington diseases as well as Alzheimer’s disease (AD) neuropathology [6], [7]. It is involved in the axonal protection against degeneration [8] and protection in models of neuronal damage based on hyperphosphorylation of the tau protein [7]. On the other hand, cell culture experiments under acute neurotoxic stress showed a dramatic reduction of SIRT1 similar to what has been observed in senescence mice models [9]. The deacetylase activity of SIRT1 also promotes memory and normal cognitive function through a mechanism distinct from the one described for the neuroprotective activity [10], [11]. Finally, the SIRT1 activator resveratrol (RES) which is a phytocompound found in red wine, was also reported to have neuroprotective effects *in vitro* and *in vivo* against oxidative stress and the amyloid-β peptide (Aβ) toxicity [12].

We report here that SIRT1 regulates dendritic development and increased dendritic arborization in hippocampal neurons. SIRT1 overexpression was sufficient to change dendritic morphogenesis and enhance dendritic arborization at early stages of development, while interfering with the catalytic deacetylase activity of SIRT1 reduced the number of dendritic branches. Furthermore, RES mimics the effect of SIRT1 on dendritic arborization at early stages of development an effect that was not observed in neurons overexpressing the dominant negative form of SIRT1. Hippocampal neurons overexpressing SIRT1 are more resistant against the cytotoxic damage induced by Aβ peptide, and avoid neuritic dystrophy. This effect depends on the deacetylase activity of SIRT1, which has a role in the maintenance of dendritic branching in a Rho kinase-dependent manner.

**Figure 1:**
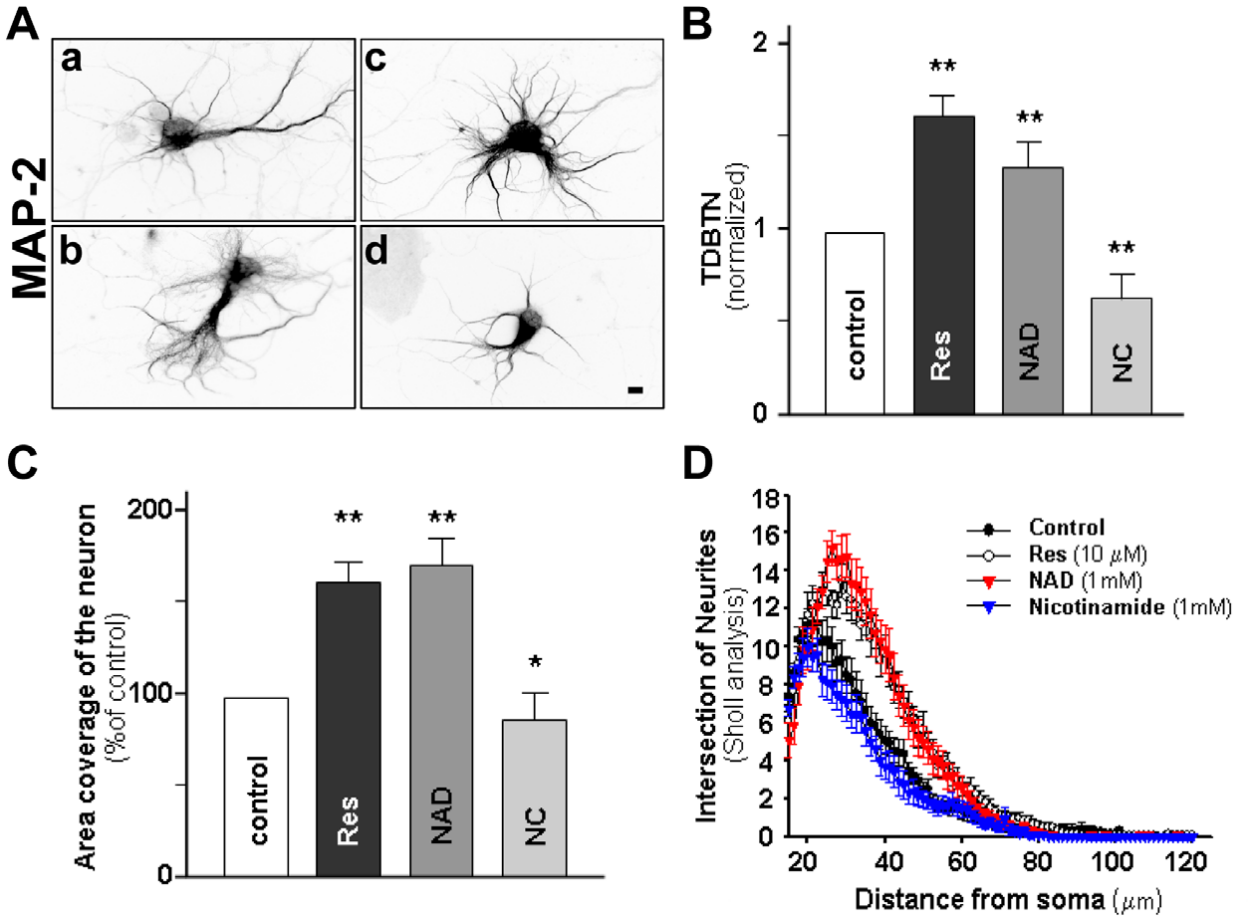
Effect of modulators of SIRT1 in neuronal dendritic branching. Hippocampal neurons at 6 days *in* vitro (DIV) were treated for 24 h with 10 µM resveratrol (Res), 1 mM NAD^+^ or 1 mM nicotinamide (NC). Neurons were fixed and MAP-2 immunostained for dendrite counting at 7 DIV. (**A**) Representative images of MAP-2 immunostaining in control neurons (**a**), and neurons treated with Res (**b**), NAD^+^ (**c**), NC (**d**). Scale bar: 10 µm. (**B, C**) Quantification of total dendritic branch tip number (TDBTN) (B), and covertures area of neurons (C) in all conditions normalized to control neurons. (**D**) Sholl analysis control neurons (black circles), and neurons treated with RES (white circle), NAD^+^ (red triangle) or nicotinamide (blue triangle). Resveratrol and NAD^+^ treatment significantly increased the number of intersection at 23–45 µm from the some compared to control neurons (p<0.01 at 23–45 µm). Neurons treated with nicotinamide did not change the intersection number compared to control. Statistical analysis was performed by Kruskal-Wallis/Dunn. n = 50 neurons. Error bars indicate S.E.M. *p<0.05; **p<0.01.

**Table 1:** Effect of resveratrol, NAD+ or nicotinamide in the number of primary and secondary dendrites.

Dendrites	Control	Resveratrol 10µM	NAD+1 mM	Nicotinamide 1 mM
**Primary**	11.4±0.9	15.0±0.8*	16.0±0.9*	8.4±0.7**
**Secondary**	4.8±0.7	7.3±0.7*	6.7±0.8*	3.1±0.7**

* p*<*0,01,

** p<0,05.

A partial account of this work was presented at the Society for Neuroscience Annual Meeting 2010 [13].

## Materials and Methods

### Reagents

Resveratrol and NAD^+^ were obtained from Sigma-Chemical, St. Louis, MO; Hoescht from Invitrogen (Carlsbad, CA); Y-27632 (4-[(1R)-1-aminoethyl]-N-pyridin-4-yl-cyclohehaxe-1-carboxamide), SP600125, TAT-TI-JIP were obtained from Calbiochem (Darmstadt, Germany), rabbit anti-SIRT1 [14], β-catenin (Santa Cruz Biotechnology Inc., Santa Cruz, CA), anti-acetyl histone H3 (Milipore Billerica, MA) antibodies, secondary antibodies labelled with 488Alexa, 543Alexa or 633Alexa (Affinity Bio Reagents Inc., Golden, CO). Aβ_1−40_ peptides corresponding to the human sequence (Bachem Inc., Torrance, CA, lot no. T-20964amd and Genemed Synthesis Inc., South San Francisco, CA) were dissolved in dimethyl sulphoxide (DMSO) at a concentration of 15 µg/µl and immediately stored in aliquots at −20°C before assaying.

**Figure 2:**
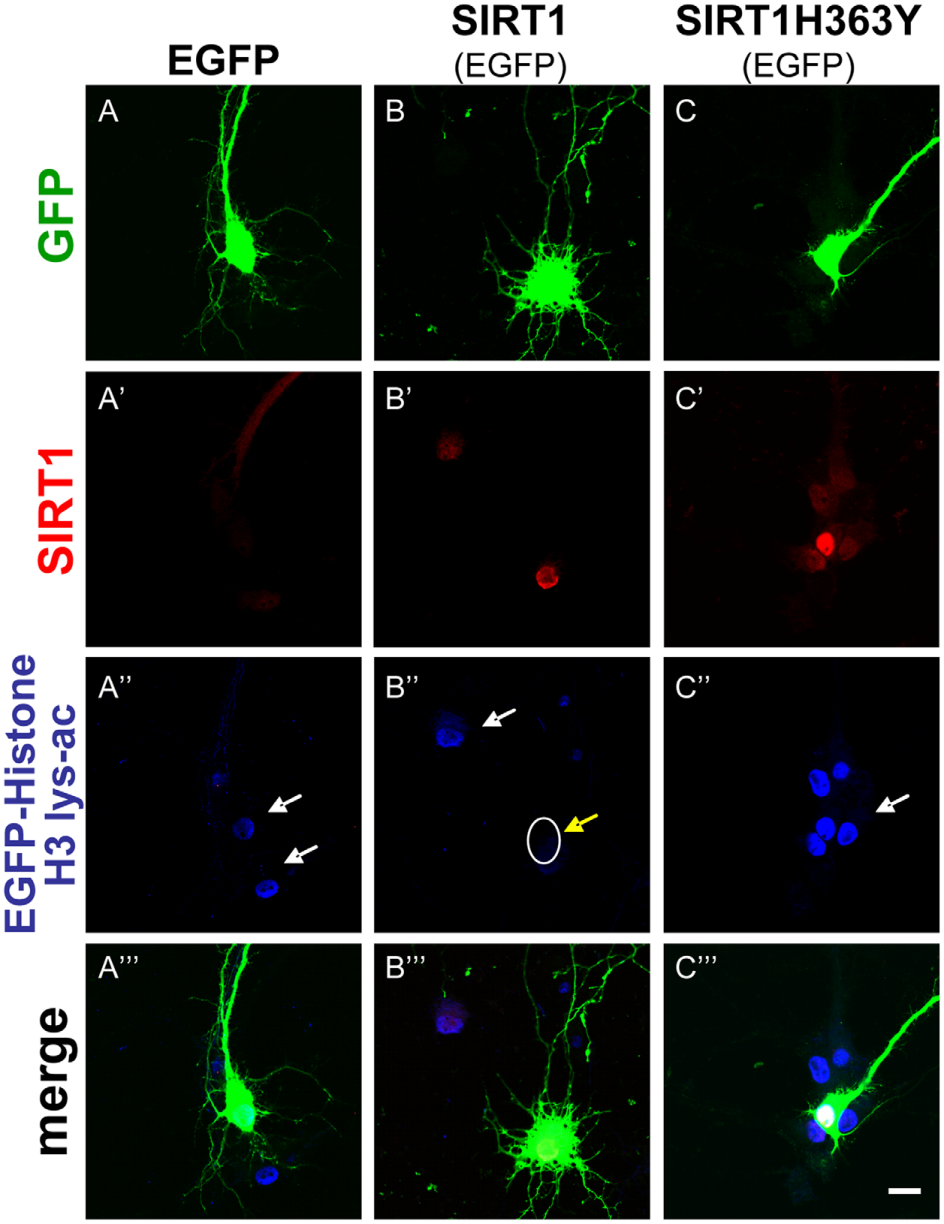
SIRT1 deacetylase activity is present in Hippocampal neurons. Hippocampal neurons were transfected at 3 DIV with GFP, SIRT1/GFP or SIRT1H363Y/GFP. Representative images are shown of the immunodetection of SIRT1 in control neurons transfected only with GFP (A’), transfected with SIRT1/GFP (B’) or SIRT1H363Y/GFP (C’). SIRT1 deacetylase activity was detected with a specific antibody to detect acetylated histone H3 in control neurons (A’’, white arrow), SIRT1 positive neurons (B’’, acetylated histone H3: white arrow and desacetylated histone H3 :white circle with yellow arrow) and neurons transfected with SIRT1H363Y (C’’, acetylated histone H3: white arrow). GFP positive cells (A, B and C) and merged images are also shown for the three experimental conditions (A’’’, B’’’ and C’’’). Scale bar, 10 µm.

**Table 2:** Effect of SIRT1 in primary and secondary dendrite number.

	RES (µM)	Primary	Secondary
**GFP**	**0**	12.8±0.8	4.8±0.6
	**1**	11.1±0.8	6.4±0.5
	**10**	16.4±0.9*	7.1±0.7*
	**50**	15.8±1.1*	9.8±0.6*
**SIRT1**	**0**	18.6±1.1*	9.1±0.8*
	**1**	17.0±1.3*	9.3±0.5*
	**10**	19.0±1.0*	13.4±0.7*
	**50**	18.3±1.0*	16.7±1.1*
**SIRT1H363Y**	**0**	7.1±0.7*	2.4±0.6*
	**1**	6.5±1.0*	2.7±0.8*
	**10**	6.0±0.8*	2.9±0.5*
	**50**	6.1±0.5*	3.0±0.5*

* p<0.01.

### Ethics Statement

Sprague-Dawley rats used in these experiments were housed at the Faculty of Biological Sciences of the P. Universidad Católica de Chile and handled according to guidelines outlined and approved by the Institutional Animal Care and Use Committee at the Faculty of Biological Sciences of the P. Universidad Católica de Chile. Animals were euthanized by anesthesia overdose.

**Figure 3:**
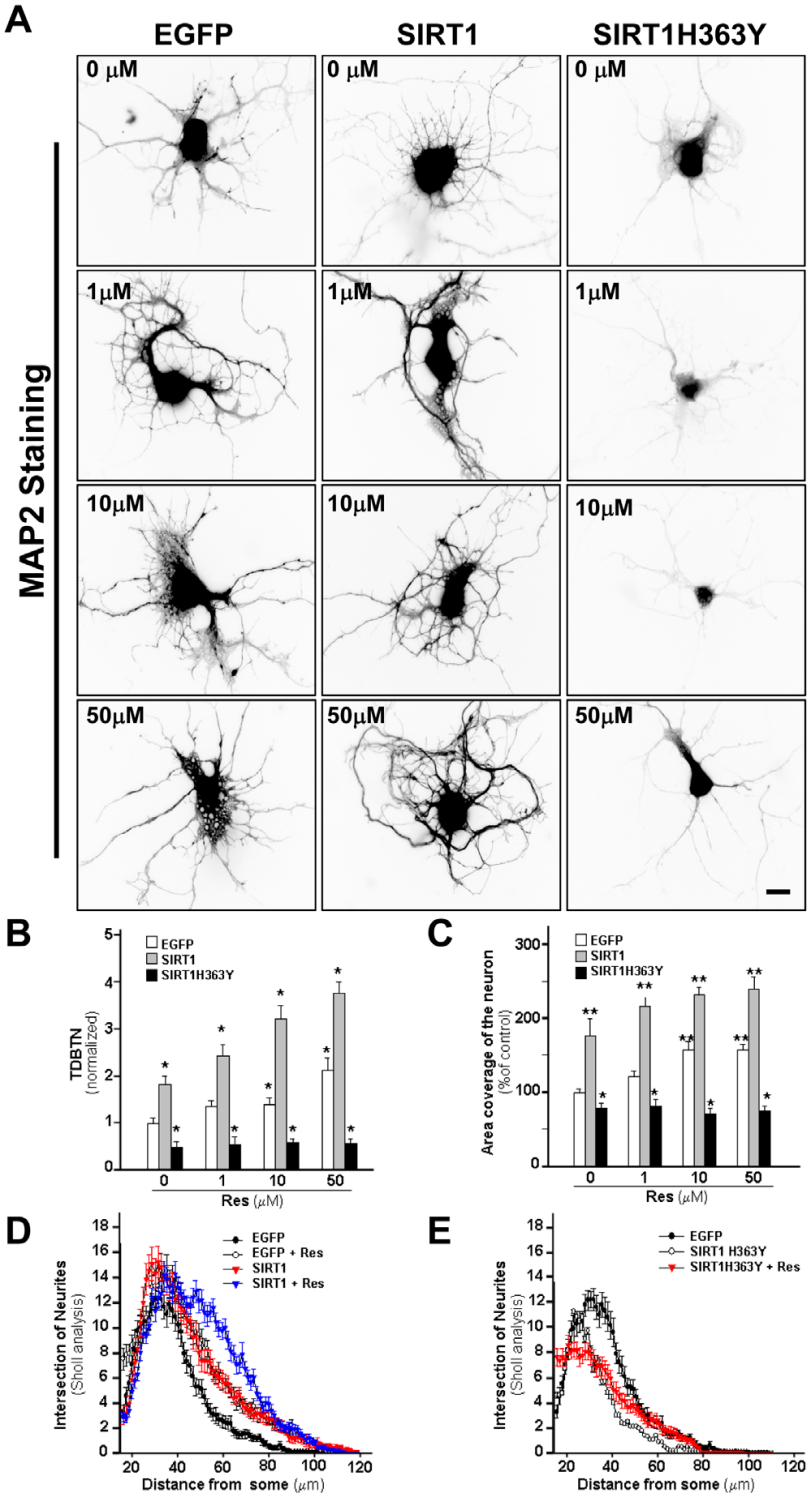
SIRT1 deacetylase activity regulates dendritic arbor complexity in hippocampal neurons. Neurons at 3 DIV were transfected with EGFP, SIRT1/EGFP or SIRT1H363Y/EGFP, then were treated at 4 DIV with Res (1, 10 or 50 µM), fixed at 6 DIV and immunostained against MAP-2 for dendrite analysis. (**A**) Representative images of control neurons transfected with EGFP (left column), SIRT1/EGFP (middle column) or SIRT1H363Y/EGFP (right column) and treated with different concentration of Res. Scale bar, 10 µm. (**B, C**) Quantification of TDBTN (B), and coverage area (C) in the presence or absence of different concentrations of Res (1, 10 or 50 µM). Ten fields were randomly selected photography. Values were normalized against control neuron transfected with EGFP. (**D**) Sholl analysis of neurons tansfected with GFP (black circles), GFP + Res 10 µM (white circle), SIRT1/GFP (red triangle) or SIRT1/GFP + Res 10µM (blue triangle). Both, Res treatment and SIRT1 transfection significantly increases the number of intersection at 25–80 µm from the soma (p<0.01 at 25–80 µm) and a higher increase in the number of intersection at 40–90 µm from the some (p<0.001 at 40–90 µm). (**E**) Sholl analysis of neurons transfected with the dominant negative of SIRT1. EGFP (black circles), SIRT1H363Y/EGFP (white circle), SIRT1H363Y/EGFP + Res (red triangle). SIRT1H363Y/EGFP transfection significantly decreased the number of intersection at 28–45 µm from the soma compared to control EGFP trasfected neurons (p<0,01 at 28–45 µm), and it prevented the effect of Res. Experiments were made triplicate and *p* values were determined by Kruskal-Wallis/Dunn. Error bars indicate S.E.M. n = 50–60 neurons. *p<0.01; **p<0.001.

**Figure 4:**
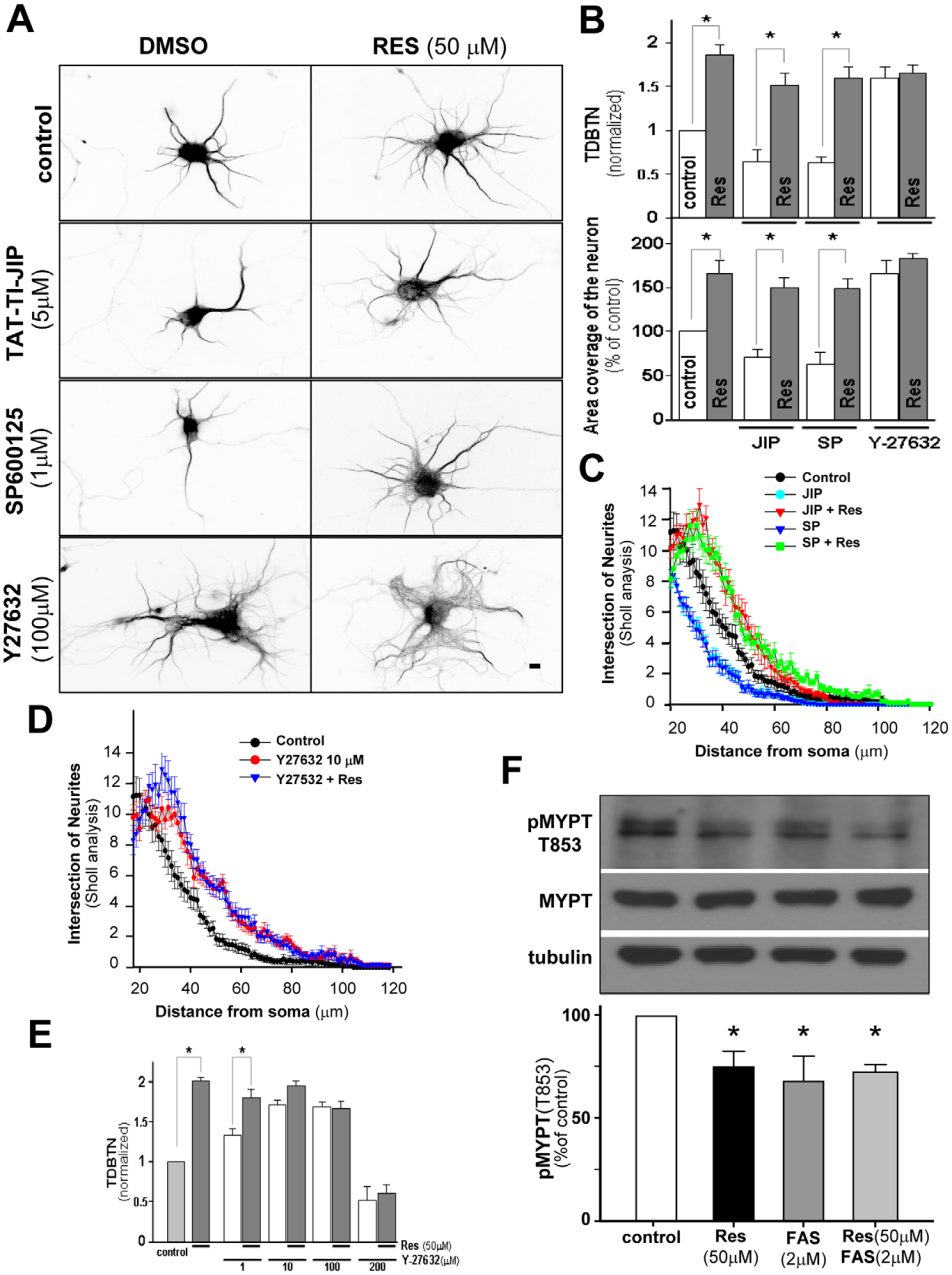
ROCK activity is involved in the regulatory effect of RES on dendrite development. Hippocampal neurons were treated at 3 DIV with 5 µM TAT-TI-JIP, 1µM SP600125 or 100 µM Y-27632 in the presence or absence of 50 µM RES. Neurons were fixed at 6 DIV and immunostained against MAP-2. (**A**) Representative images of MAP-2 immunostaining in all experimental conditions. Scale bar, 10 µm. (**B**) Quantification of TDBTN and coverage area of neurons in all conditions shown in (A). Values were normalized against control neurons. (**C**) Sholl analysis of control neurons (black circles), and neurons treated with TAT-TI-JIP (cian circle), TAT-TI-JIP + RES (red triangle), SP600125 (blue triangle), SP600125+RES (green square), RES treatment reverts the JIP and SP600125 effect significantly increasing the number of intersection at 26–50 µm from the soma compared to non treated neurons (p<0,01 at 26–50 µm). Error bars indicate S.E.M. n = 50–60 neurons. (**D**) Sholl analysis of control neurons (black circles), and neurons treated with Y−27632 (red circle), Y−27632+ RES (blue triangle). Treatment with Y−27632 in the presence or absence of RES significantly increases the number of intersection at 24–70 µm from the some compared with non treated neurons (p<0,01 at 24–70 µm). (**E**) Quantification of TDBTN in neurons treated with different doses of Y−27632 in the presence or absence of Res. (**F**) Representative immunoblot against total Myosin Phosphatase Target 1 (MYPT1) and MYPT 1 phosphorylated at T853 (pMYPT), a specific substrate of ROCK kinase. Densitometric values of pMYPT1 bands were normalized against total MYPT1, and then each condition expressed as relative to the control condition without treatment. Experiments were carried out in triplicate. Students *t*-test, *p<0.01.

**Figure 5:**
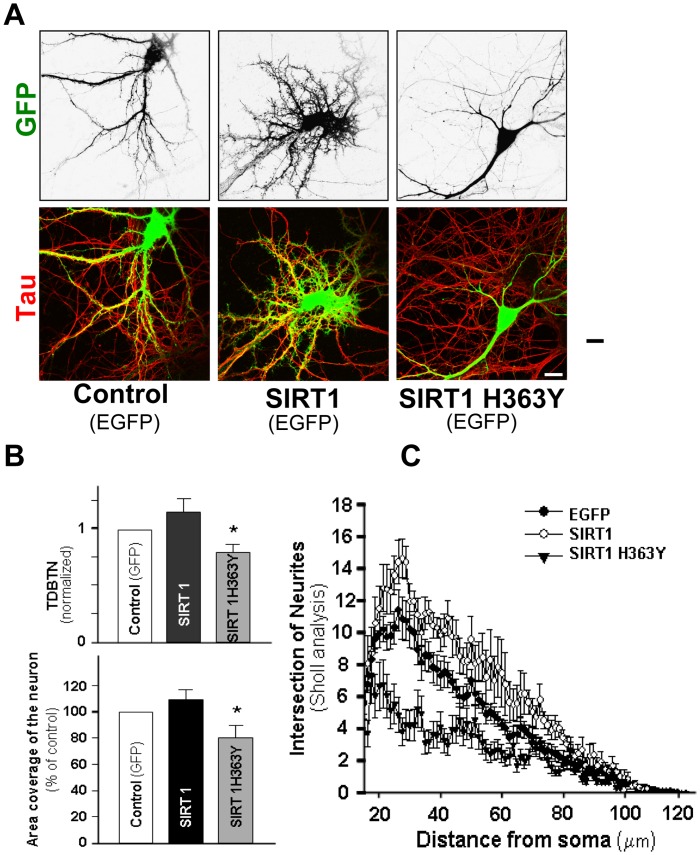
SIRT1 regulates the maintenance of the dendritic tree. Hippocampal neurons at 10 DIV were transfected with GFP, SIRT1/EGFP or SIRT1H363Y/EGFP. Neurons were fixed at 14 DIV and immunostained against Tau protein. (**A**) Representative images of Tau immunodetection in trasfected GFP positive neurons. Scale bar, 10 µm. (**B**) Quantification of TDBTN and coverage area in transfected neurons. (**C**) Sholl analysis of neurons tansfected with GFP (black circles), SIRT1/GFP (white circle), or SIRT1H363Y/GFP (black triangle). The number of intersections does not change in neurons transfected with SIRT1 compared to control GFP neurons. SIRT1H363Y/GFP transfection significantly decrease the number of intersection at 18–45 µm compared to control neurons (p<0.01 at 18–45 µm). Error bars indicate S.E.M. Experiments were made in triplicate n = 50 neurons. *p* values were determined by Kruskal-Wallis/Dunn. *p<0.05.

**Figure 6:**
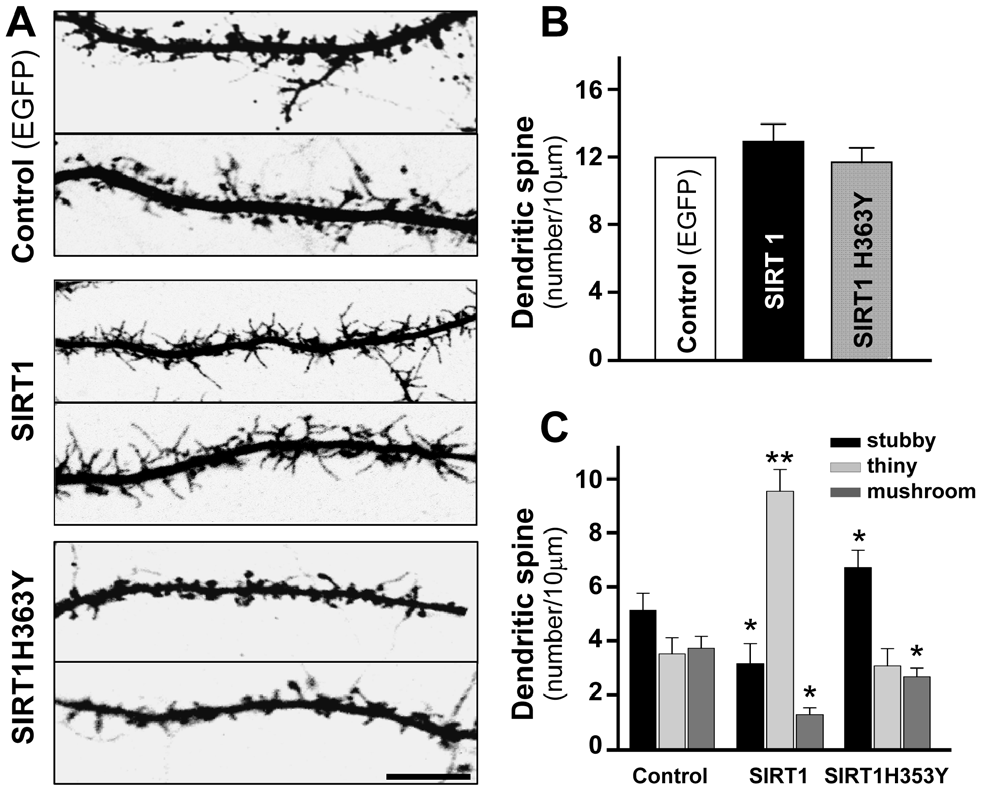
The effect of SIRT1 expression on dendritic spine density. (**A**) Hippocampal Neurons were transfected at 10 DIV and analyzed at 21 DIV. Representative images of dendritic spines in neurons expressing GFP, SIRT1/GFP or SIRT1H363Y/GFP. (**B**) Dendritic spine density (per 10 µm) in transfected neurons at 21 DIV. (**C**) Density of the different kinds of dendritic spines (per 10 µm) in transfected neurons. *p* values were determined by Kruskal-Wallis/Dunn. Error bars indicate S.E.M. n = 40 neurons. *p<0.01; **p<0.001.

**Figure 7:**
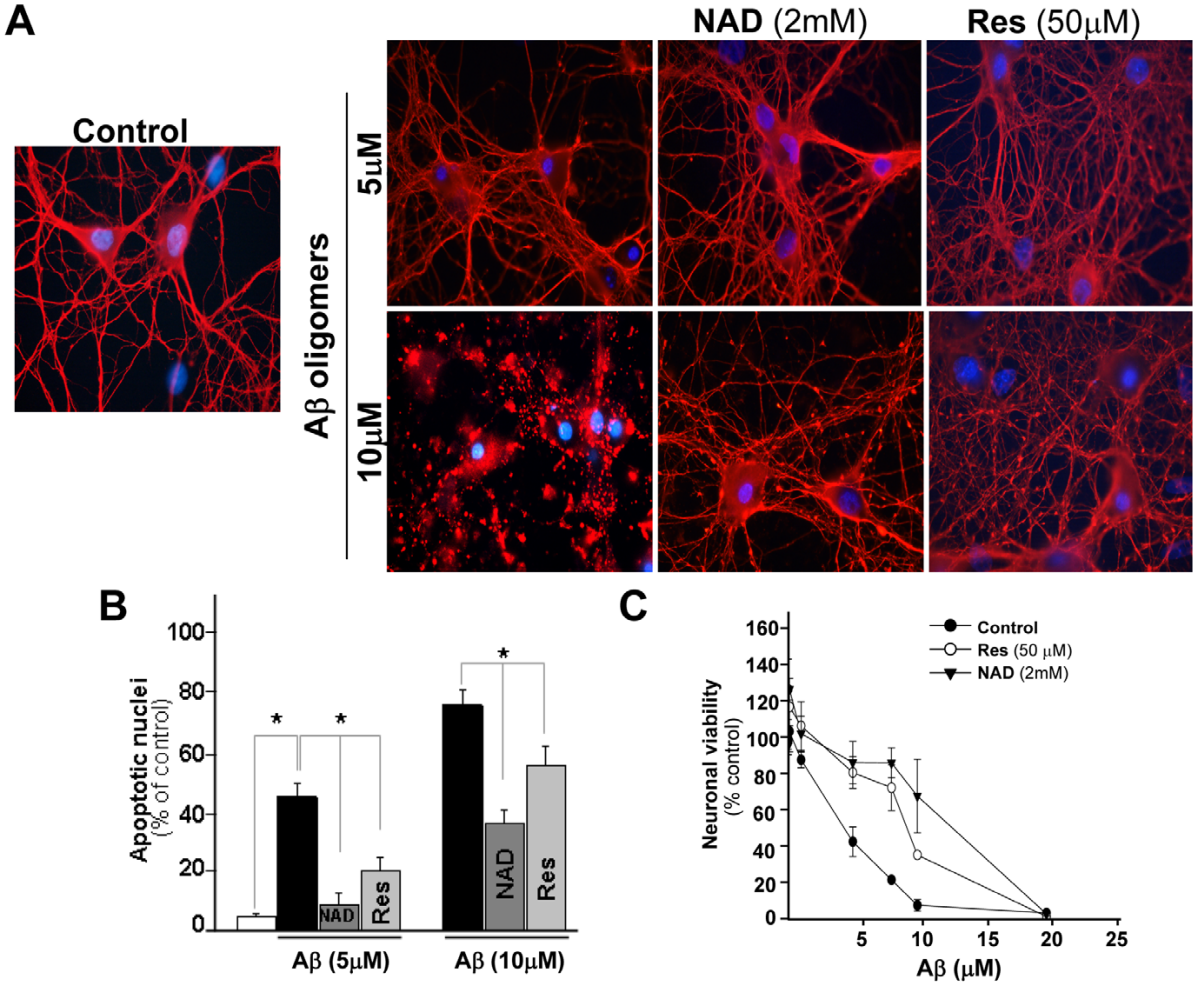
NAD+ and RES protect neurons from toxicity produced by Aβ aggregates. Hippocampal neurons at 21 DIV were treated with 5 and 10 µM Aβ oligomers for 12 h in the presence or absence of 50 µM RES or 2 mM NAD^+^. (**A**) Representative images of control and treated neurons inmunostained against Tau (red) and Hoeschst (blue). Scale bar 10 µM. (**B**) Percentage of apoptotic nuclei in neurons in the different experimental conditions. (**C**) MTT assay of hippocampal neurons treated with different concentration of Aβ fibrils with or without RES or NAD^+^ and *p<0.01.

**Figure 8:**
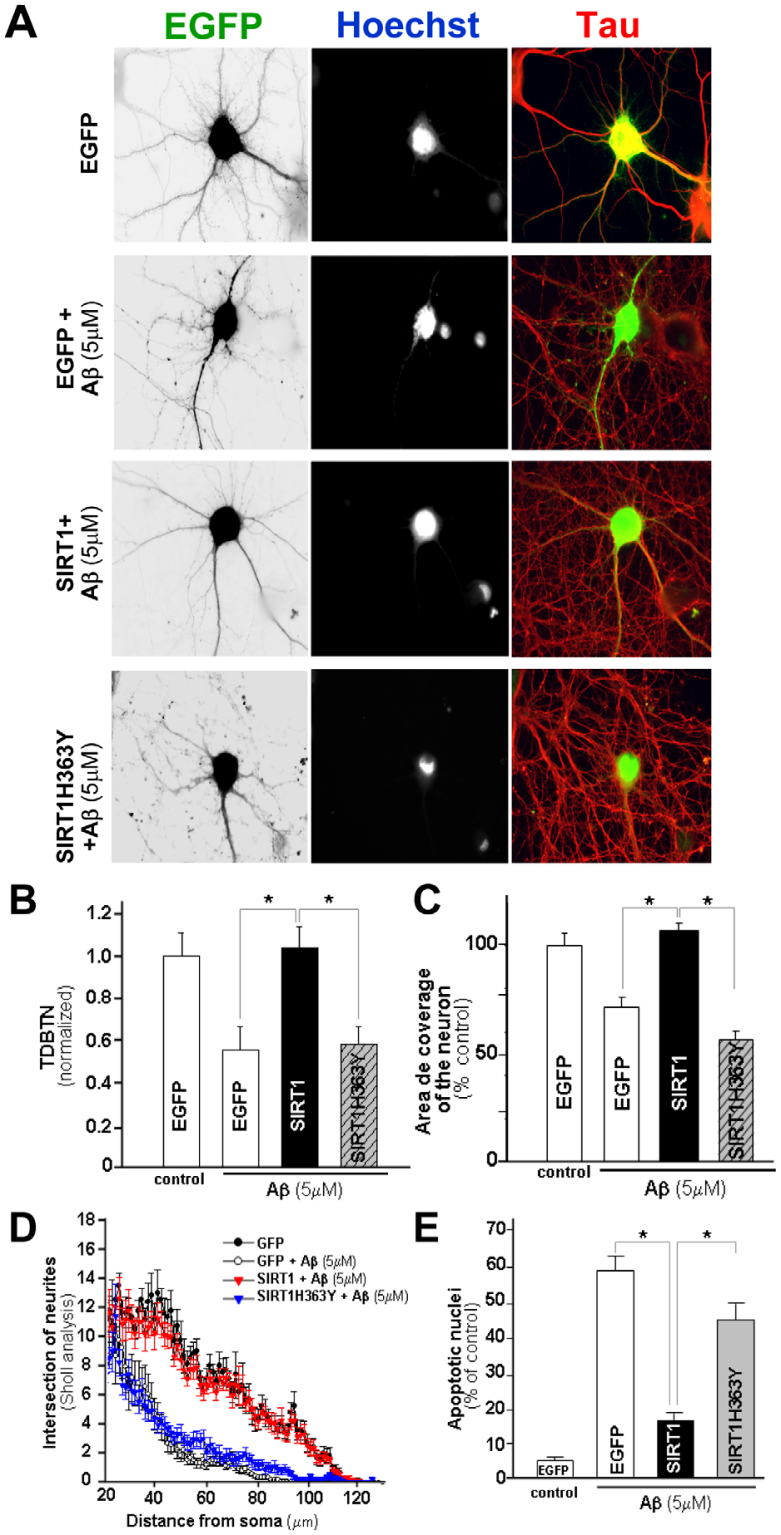
SIRT1 preserves dendritic integrity in hippocampal neurons challenged with Aβ fibrils. Hippocampal neurons at 10 DIV were transfected with EGFP, SIRT1/EGFP or SIRT1H363Y/GFP and then treated at 14 DIV with 5 µM Aβ for 12 h and then fixed and immunostained against Tau to analyze dendrites. (**A**) Representative images of EGFP transfected neurons with or without treatment with Aβ 5 µM, and neurons transfected with SIRT1/GFP or SIRT1H363Y/GFP challenged with Aβ. Scale bar, 10 µm. (**B,C**) Normalized TDBTN (B) and coverage area (C) of experimental conditions shown in (A). (**D**) Sholl analysis of transfected neurons: GFP (black circles), GFP + Aβ 5 µM (with circle), SIRT1/GFP + Aβ 5 µM (red triangle), SIRT1H363Y/GFP + Aβ 5 µM (blue triangle). Treatment with Aβ fibrils significantly decrease the number of intersection at 28–110 µm from the soma compared with GFP transfected neurons (p<0,01 at 28–100 µm). (**E**) Percentage apoptotic nuclei under the different condition assayed. *p* values were determined by Kruskal-Wallis/Dunn (*p<0.01). Error bars indicate S.E.M. n = 40 transfected neurons.

### Primary Cultures of Rat Hippocampal Neurons and Transfection

Hippocampal neurons were obtained from 18-day-old Sprague-Dawley rat embryos. Hippocampi were aseptically dissected and trypsinized for 20 min. After centrifugations for 1 min, cells were seeded in phenol red-free Dulbecco's modified Eagle's medium plus 10% horse serum into 1% poly-L-lysine-coated plates. After 120 min, medium was removed, and Neurobasal medium was added containing 1% B27 supplement from Invitrogen, plus streptomycin and penicillin. On day 3 of culture, hippocampal neurons were treated with 2 µM 1-β-D-arabinofuranosylcytosine (AraC) for 24 h to reduce the number of proliferating non neuronal cells [15]. Transfection was performed using the calcium phosphate method for neurons as described previously [16] with some modifications. Hippocampal neurons were transfected at 3 or 10 days *in vitro* (DIV 3, DIV10), and experiments were performed at 6 or 14 DIV. Constructs used for the transfection were: green fluorescent protein (GFP) from Clontech (Mountain View, CA), the SIRT1 and SIRT1 H363Y which were a kind gift from Dr. Tso-Pang Yao (Duke University, Durham, NC) and Dr. Jeff Milbrandt (Washington University Medical School, St. Louis, MO).

### Aβ Aggregation

The amyloid aggregates were obtained as previously described [17], [18]. Briefly, stock solutions were prepared by dissolving freeze-dried aliquots of Aβ in Me_2_SO. Peptide stock aliquots were diluted in 0.1 M Tris-HCl (pH 7.4) to a final concentration of 100 µM Aβ. The solutions were stirred continuously (210 rpm) at room temperature for 24 hrs to obtain fibrils or 1 h to obtain oligomers.

### Cell Viability Assay (MTT Reduction)

MTT assays were performed as described previously [19], [20]. Hippocampal neurons (2×10^4^ cells/100 µl/well) were assayed in B27- and phenol red-free medium. Neurons were pre-incubated for 2 h with 20 µM of resveratrol, 2 mM of NAD+ or medium (control). Then, different concentrations of Aβ aggregates was added. Cells were incubated for 12 h at 37°C, after which cell viability was measured by the MTT method. MTT reduction was determined in a Lab Systems Uniskam I spectrophotometer at 540 and 650 nm.

### Immunofluorescence

Hippocampal neurons were plated on polylysine-coated coverslips (30,000 neurons/cover). After 3 DIV neurons were transfected and then fixed at DIV 6 for dendrite analysis. For transfected neurons, transfection was carried out at 10 DIV and fixation at 14 DIV for dendrite analysis or exposed to Aβ fibrils. Cells were rinsed twice in ice-cold PBS and fixed with a freshly prepared solution of 4% paraformaldehyde in PBS for 20 min and permeabilized for 5 min with 0.2% Triton X-100 in PBS. After several rinses in ice-cold PBS, cells were incubated in 1% BSA in PBS (blocking solution) for 30 min at room temperature, followed by an overnight incubation at 4°C with primary antibodies. Cells were extensively washed with PBS and then incubated with Alexa-conjugated secondary antibodies (Molecular Probes, Carlsbad, CA, USA) for 30 min at 37°C. Primary antibodies used were monoclonal anti-MAP-2 (Santa Cruz Biotechnology Inc., Santa Cruz, CA) and polyclonal anti-Tau (DAKO, Denmark). For dendritic spines analyses, z-stacks were acquired with a 100× oil immersion Plan-Apochromat objective.

### Sholl Analysis

Sholl analysis was performed with a semiautomated program, in which the soma boundary is approximated by an ellipsoid and dendrite intersections were assessed at radial distances from the soma [21]. The dendritic tree was examined in 5 µm increments. Statistical analysis was done with ANOVA followed by the appropriate post hoc test.

### Immunoblot Analysis

Neurons grown on 6-well culture plates were lysed in ice-cold lysis buffer (10 mM Tris-HCl, pH 7.8, 100 mM NaCl, 10 mM EDTA, 0.5% Nonidet P-40, and 0.5% sodium deoxycholate) supplemented with protease and phosphatase inhibitors. Homogenates were maintained in ice for 30 min and then centrifuged at 10,000 rpm for 10 min (4°C). The supernatant was recovered and protein concentration was determined by BCA protein assay kit (Pierce, Rockford, IL, USA). Proteins were resolved in SDS-PAGE (8% polyacrylamide), transferred to PVDF membrane and reacted with primary antibodies. The reactions were followed by incubation with secondary antibodies peroxidase labeled (Pierce) and developed using the ECL technique (PerkinElmer, Waltham, MA). Primary antibodies used were: mouse monoclonal anti-MYPT1, 1∶1000 (Becton Dickinson, NJ USA), rabbit polyclonal anti-phospho MYPT1 Thr 853, 1∶1000 (Cyclex, Nagano Japan) and mouse monoclonal anti-α-tubulin 1∶80.000 (Sigma, MO USA).

## Results

### Resveratrol and NAD^+^ Increase Dendritic Branching Complexity

In the embryonic rat brain, SIRT1 is expressed in the ventricular and subventricular zones where SIRT1 has a role in neuronal differentiation [5]. To address the potential role of SIRT1 during differentiation of hippocampal neurons, dendrite development was assessed in neurons treated with RES (a SIRT1 activator), NAD^+^ (a SIRT1 substrate) and Nicotinamide (an inhibitor of SIRT1). Treatment was carried out at 3 days *in vitro* (DIV), and neurons were analyzed at 6 DIV. Control untreated neurons show a typical pyramidal morphology with several short dendrites and in the presence of RES or NAD^+^, neurons showed longer dendrites with increased number of dendritic branches and increased complexity (Figure 1A). Both treatments, RES and NAD^+^, increased total dendritic branch tip number (TDBTN) (Figure 1B) compared to control neurons, while Nicotinamide significantly decreased TDBTN (control: 1±0.11; RES: 1.6±0.1; NAD^+^: 1.4±0.1; Nicotinamide: 0.6±0.1). Treatment with RES and NAD^+^ also increased primary and secondary dendrites (Table 1) and consequently the coverage area of neurons (Figure 1C), which was decreased by Nicotinamide treatment (control: 100% ±9.7; RES: 157.3% ±8.6; NAD^+^: 164.4% ±8.8; Nicotinamide: 80.2% ±7.0). Sholl analysis indicates that there is an increase in dendritic arbor complexity after treatment with RES and NAD^+^, while treatment with Nicotinamide induced no changes in the branch order of dendrites (Figure 1D). RES and NAD^+^ treatment significantly increases number of intersection at 23–45 µm from the soma compared with non treated neurons. These results strongly suggest that SIRT1 may regulate dendritic development.

### SIRT1 Regulates Dendrite Growth in Hippocampal Neurons

To further characterize the potential role of SIRT1 in dendrite development, hippocampal neurons were co-tranfected at 3 DIV with GFP and a vector coding for SIRT1 or its mutant version SIRT1H363Y that lacks the catalytic activity acting as a dominant negative of SIRT1 deacetylase [22], [23]. As expected, neurons overexpressing SIRT1 or SIRT1H363Y showed an increased immunoreactivity against SIRT1 in their nuclei (Figure 2B’ and 2C’) as compared to control neurons transfected only with GFP (Figure 2A’), since the polyclonal antibody anti-SIRT1 recognizes both, the wild-type and mutant versions of SIRT1. However, only neurons overexpressing wild-type SIRT1 show increased deacetylase activity as determined by decreased levels of acetylated histone H3 (Figure 2B’’), one of the targets of deacetylase activity of SIRT1 [24], [25], as compared to control neurons (Figure 2A’’) and neurons overexpressing SIRT1H363Y (Figure 2C’’).

Dendritic arborization was analyzed in transfected neurons at 6 DIV, in the presence or absence of increasing concentrations of RES. Control neurons transfected only with GFP showed a typical pyramidal morphology with long axons and several short dendrites (Figure 3A). GFP positive neurons in the presence of RES, as well as, neurons overexpressing SIRT1 show higher dendritic arbor complexity (Figure 3A), with increased number of primary and secondary dendrites (Table 2). On the contrary, neurons transfected with SIRT1H363Y show a strong decrease in dendritic arborization even in the presence of RES (Figure 3A, Table 2). SIRT1 overexpression strongly increased TDBTN (Figure 3B), while overexpression of the dominant negative form of SIRT1 significantly decreased TDBTN (GFP: 1.0±0.1; SIRT1/GFP: 1.8±0.2; SIRT1H363Y/GFP: 0.5±0.1). Treatment of control GFP transfected neurons with RES increased TDBTN in a dose-dependent manner (1 µM: 1.3±0.1; 10 µM: 1.5±0.1; 50 µM: 2.1±0.3), and there is a stronger effect of RES in SIRT1/GFP transfected neurons (1 µM: 2.4±0.2; 10 µM: 3.2±0.3; 50 µM: 3.8±0.3). Interestingly, the effect of RES was completely prevented in neurons transfected with SIRT1H363Y/GFP (1 µM: 0.6±0.2; 10 µM: 0.6±0.1; 50 µM: 0.6±0.2), indicating that RES increases dendritic arbor complexity via the deacetylase activity of SIRT1 (Figure 3B). Accordingly, SIRT1 overexpression and RES treatment increased dendritic coverage area, while SIRT1H363Y overexpression prevented the effect of RES (Figure 3C). Sholl analysis indicates that there is an increase in dendritic arbor complexity in neurons overexpressing SIRT1 as well as in neurons treated with 10 µM RES (Figure 3D). SIRT1 transfection significantly increases the number of intersection at 25–80 µm from the soma compared to control GFP transfected neurons (p<0.01 at 25–80 µm), and an additive effect was observed in neurons overexpressing SIRT1 and treated with 10 µM RES (Figure 3D), which show increased number of intersections at 40–90 µm from the soma compared to control neurons (p<0.001 at 40–90 µm). On the other hand, neurons transfected with the SIRT1H363Y construct showed decreased dendrite branching as determined by Sholl analysis and strongly decreased the effect of RES (Figure 3E). These results indicate that the deacetylase activity of SIRT1 is implicated in the development of the dendritic arbor in hippocampal neurons.

### The Effect of RES on Dendrite Morphogenesis Involves ROCK Activity

To further elucidate the signaling pathway downstream of SIRT1, we studied the contribution of the Jun-terminal kinase (JNK), a downstream target of Rac [26], [27]. We used SP600125 a specific JNK inhibitor as well as, a small peptide TAT-TI-JIP that inhibits JNK and it is structurally unrelated to SP600125 [28]. Neurons treated with SP600125 or TAT-TI-JIP decreased dendritic arbor complexity, but these inhibitors were not able to prevent the effect of RES (Figure 4A). This is clearly shown in the TDBTN quantification and the coverage area of neurons (Figure 4B) and in the number of intersections (Figure 4C), which were strongly induced by RES even in the presence of SP600125 and TAT-TI-JIP, suggesting that RES activates dendritogenesis by a JNK-independent pathway.

Then, we tested the contribution of Rho GTPases in dendritic development. We first assayed the contribution of RhoA by inhibiting its downstream effector Rho-associated protein kinase (ROCK), using the specific ROCK inhibitor Y−27632 [29], [30]. Neurons treated with Y−27632 showed increased morphology complexity (Figure 4A), with increased TDBTN and coverage area (Figure 4B, TDBTN: control: 1±0.1; Y−27632∶1.6±0.2; Coverage area: control: 100±9.7; Y−27632∶160.0±11.2). Treatment with Y−27632 in the presence of RES did not further increase TDBTN (Y−27632+ RES: 1.6±0.2), or coverage area of neurons (Y−27632+ RES: 180.0±9.8). The same occurs with the number of intersections (Figure 4E), suggesting that RES modulates the complexity of the dendritic arbor trough the inhibition of ROCK. Treatment with RES was carried out in the presence of different doses of Y−27632 from 1 to 200 µM. A concentration-dependent increase in TDBTN was observed by Y−27632 treatment (Figure 4E), however none of the concentrations of Y−27632 was able to further increase the effect of RES supporting that the same pathway is involved in the effect of Y−27632 and RES, and therefore that ROCK inhibition is involved in the effect of RES.

Finally, and to evaluate whether RES modulate ROCK activity, we evaluated the phosphorylation state of myosin phosphatase targeting protein 1 (MYPT1, also known as Myosin Binding Subunit MBS) in its residue T853, an exclusive target of ROCK [31]. Hippocampal neurons were treated for 24 h with 50 µM RES in the presence or absence of 2 µM Fasudil. At this concentration, Fasudil is a potent and selective inhibitor of ROCK [30], [32]. As shown in Figure 4F, treatment with RES and Fasudil decreased the levels of phosphorylated MYPT1. The same effect was observed by co-treatment with RES plus Fasudil. This result strongly support that RES acts through inhibition of ROCK.

### SIRT1 Deacetylase Activity Regulates the Maintenance of the Dendritic Arbor

To examine if the regulatory effect of SIRT1 in dendritic morphogenesis is restricted to early stages of development, neurons were transfected with GFP, SIRT1/GFP or SIRT1H363Y/GFP at 10 DIV and analyzed at 14 DIV (Figure 5A). Overexpression of SIRT1 did not result in a significant change in TDBTN (GFP: 1±0.1; SIRT1/GFP: 1.2±0.1), dendritic coverage area (GFP: 100±8.5%; SIRT1/GFP: 114.7±4.5%), or arbor complexity compared to control neurons (Figure 5B and 5C). However, overexpression of the catalytically inactive SIRT1H363Y decreased TDBTN (SIRT1H363Y/GFP: 0.82±0.07) and coverage area of neurons (SIRT1H363Y/GFP: 82.6±6.6%), and significantly decrease the number of intersection at 18–45 µm compared to control neurons (p<0.01 at 18–45 µm) suggesting the deacetylase activity of SIRT1 also regulates the maintenance of the denritic arbor (Figure 5A–C).

### SIRT1 Activity Regulates the Density of Dendritic Spines in Hippocampal Neurons

Dendritic spines play a major role in memory acquisition and LTP [33]. Number, size and shape of dendritic spines are critical parameters to determine neuronal function and provide the structural basis for synaptic plasticity. To assess the potential role of SIRT1 on dendritic spines morphogenesis, neurons were transfected at 10 DIV with GFP, SIRT1/GFP or SIRT1H363Y/GFP and analyzed at 21 DIV (Figure 6A). For this analysis, dendritic spines were classified into 3 classes: (1) mushroom spines: the diameter of the head is much higher than the diameter of the neck; (2) stubby spines: the diameter of the neck is similar to that of the head; (3) thin spines: the length is greater than the diameter, and length is less than 3 µm [34]. Although no significant changes were observed in the density of dendritic spines by SIRT1 or the dominant negative SIRT1H363Y overexpression (Figure 6B, spines per 10 µm length: GFP: 12.2±0.8; SIRT1/GFP: 13.7±0.6; SIRT1H363Y/GFP: 12.3±0.6), a strong increase in the density of thin spines and a decrease in stubby and mushroom spines were observed in neurons overexpressing SIRT1 (Figure 6C, GFP_stubby_: 5.0±0.6; GFP_thin_: 3.5±0.6; GFP_mushroom_: 3.7±0.4; SIRT1/GFP_stubby_: 3.1±0.6; SIRT1/GFP_thin_: 9.2±0.7; mushroom: 1.3±0.24). Neurons overexpressing SIRT1H363Y showed increased density of stubby and decreased number of mushroom spines (SIRT1H363Y/GFP_stubby_: 6.5±0.6; SIRT1H363Y/GFP_thin_: 3.1±0.6; SIRT1H363Y/GFP_mushroom_: 2.7±0.3). These results suggest that the catalytic activity of SIRT1 modulates spine architecture.

### SIRT1 Protects Against Neuritic Dystrophy Induced by Aβ Aggregates

Considering our findings, we next evaluate whether SIRT1 could have a protective role against dendritic damage induced by the Aβ peptide. First, we evaluated the effect of the two activators of SIRT1, RES and NAD^+^ in neurons treated with 5 and 10 µM Aβ oligomers. As determined by immunofluorescence against tau, neurons treated with 10 µM Aβ showed a strong dendritic dystrophy (Figure 7A), which is partially prevented by 20 µM RES and 2 mM NAD^+^. Apoptotic nuclei detected by Hoechst staining, was also decreased by co-treatment with RES or NAD^+^ (Figure 7B). Cell viability was also assessed by MTT assay in neurons treated with increasing doses of Aβ aggregates (0–20 µM). A dose-dependent decrease in cell viability was observed in Aβ-treated neurons, and neurotoxicity was partially prevented by co-treatment with 20 µM RES and 2 mM NAD^+^ (Figure 7C). The neuroprotection induced by RES and 2 mM NAD^+^ suggests that the activation of SIRT1 could have a neuroprotective role against Aβ neurotoxicity.

To evaluate the neuroprotective properties of SIRT1, neurons were transfected at 10 DIV with GFP, SIRT1/GFP or SIRT1H363Y/GFP and treated with 5 µM Aβ fibrills. GFP transfected neurons show an altered dendritic morphology (Figure 8A), and a decreased dendritic arbor complexity as determined by a decrease in TDBTN (GFP: 1±0.1; GFP+Aβ: 0.6±0.1), neuronal area (GFP 100% ±7.5; GFP+Aβ: 69.9% ±6.4) and intersections of neurites (Figure 8B–D). All these parameters were improved in SIRT1-transfected neurons but not in neurons transfected with the dominant negative form SIRT1H363Y (TDBTN = SIRT1/GFP+Aβ: 1.1±0.4; SIRT1H363Y/GFP+Aβ: 0.6±0.2; Coverage area = SIRT1/GFP+Aβ: 104.6% ±5.6; SIRT1H363Y/GFP+Aβ: 58.9% ±5.8). Transfection with SIRT1 was also able to prevent the increase in the number of neurons with apoptotic nuclei observed by treatment with Aβ (Figure 8E). Altogether, these results indicate that the deacetylase activity of SIRT1 could help to maintain the integrity of the dendritic tree in the presence of Aβ.

## Discussion

In this study we describe an important link between SIRT1 and dendritic growth regulation. In addition, we have demonstrated that SIRT1 not only regulates dendritic branching in hippocampal neurons, but it also maintains dendritic arbor in normal conditions as well as under neurotoxic damage of Aβ.

The rate of dendritic growth and arborization during normal development is relatively stable from 3 to 12 DIV [35]. We observed that SIRT1 overexpression induced an increase in dendritic arborization in young neurons at 6 DIV, an effect that was also observed for RES, a SIRT1 activator, and the SIRT1 substrate NAD^+^. These results strongly suggest a relevant role for the enzyme in dendrite morphogenesis. Consistent with this idea, the loss of deacetylase activity of SIRT1 or the treatment with Nicotinamide, the inhibitor of SIRT1, affected normal dendritic development. The increase of dendritic growth in mature neurons results in dendritic filopodia that are more likely to encounter near-by axons and form spines, which are also specialized actin-based structures [36]. Indeed, we observed no changes in spine density in neurons expressing SIRT1 or SIRT1H363Y. These results are consistent with previous observations made in neurons of adult SIRT1 knock-out mice, which had a severely reduced dendritic tree compared with the wild-type strain, but had no changes in the density of dendritic spines [11]. However, when analyzing the different subtypes of spines, we observed a significant change in the relative proportion of mushroom, stubby and thin spines in SIRT1 or SIRT1H363Y, in contrast to observations in the animal-SIRT1 KO. These differences could be due to dendritic analysis methods. In the study by Michán and coworkers, Golgi staining was used, which has number of drawbacks compared to other methods of dendritic analysis. For example, it underestimates spine density by a factor of 3 compared to fluorescence methods [37]. It is assumed that a portion of spines is not detected because the image is usually analyzed in 2D, therefore the spines that protrude in the z plane, are poorly detected [38]. Our results suggest that SIRT1 is relevant for spine morphology, at least at the stage of development examined.

In knock-out mice it cannot be determined whether the phenotype results from defects during development and/or in the maintenance of the dendritic tree. To this end, we have determined a positive effect of SIRT1 on dendritic development and in mature neurons at 14 DIV, the deacetylase activity of SIRT1 was shown to be required for the maintenance of the dendritic arbor since the loss of the catalytic activity decreased the complexity of the dendritic tree. On the other hand, SIRT1 overexpression in mature neurons had no effect on dendritic arborization, which may imply a differential role for SIRT1 in the development and maintenance of the dendritic arbor.

In an attempt to investigate downstream effectors that ultimately control the dynamics of the cytoskeleton and underlies the observed structural changes of dendrites, we studied the potential role of the family of Rho GTPases, which includes the proteins RhoA, Rac1 and Cdc42; these are key intracellular switches that regulate dendritic growth and maintenance [39]. In both, neuronal cell lines and primary neuronal cultures, activation of RhoA inhibits dendritic growth, while Rac1/Cdc42 activation promotes it. For this reason, it has been suggested that for the normal development and formation of functional neuronal circuits, it is required the activation of Rac1/Cdc42 simultaneous to the inhibition of RhoA [40]. As expected, pharmacological inhibition of JNK, a downstream effector of Rac1, caused a decrease in dendritic growth. Conversely, inhibition of ROCK, a downstream effector of RhoA, produced a significant increase in dendritic growth in cultured neurons. Regarding the effect of RES, we determined that the increase in dendritic growth is independent of Rac1/JNK activation, as inhibitors of this pathway did not prevent the effects of RES. On the other hand, our results suggest that inhibition of RhoA/ROCK are part of the same pathway of SIRT1 since the co-application of RES with the inhibitor of RhoA/ROCK did not produce an additive effect on dendritic growth.

In addition, we determined that RES inhibits ROCK activity in neurons, as treatment with RES decreased the levels of MYPT1 phosphorylated at threonin 853 that is a specific phosporylation target of ROCK. Morover, and in line with our previous results, co-application of RES plus the specific pharmacological inhibitor of ROCK fasudil did not produce a further inhibition of MYPT1 phosphorylation at threonin 853. Interestingly, MYPT1 phosphorylated by ROCK, inhibits the activity of Myosin Light Chain (MLC) phosphatase and thereby indirectly increases MLC phosphorylation and actomyosin contractility [40]–[42], which may deacrease dendritic arborization. Therefore, the decrease in MYPT1 phosphorylation at threonin 853 by RES could be part of the mechanism involved in the effect of RES on dendritic arborization. As a whole, our findings strongly suggest that inhibition of the RhoA/ROCK pathway, is part of the mechanism downstream the activation of SIRT1 by RES.

It has been proposed that SIRT1 activity may play an important role in neuroprotection. SIRT1 and caloric restriction show neuroprotective properties in mouse models for Alzheimer’s [7], [43] and Parkinson’s [44] diseases. SIR2 orthologs can also protect against neuronal dysfunction due to poly-glutamine toxicity in *C. elegans* and mammalian cells [45]. However, the mechanisms by which SIRT1 confers neuroprotection, are poorly understood. Previous studies in AD-type model transgenic mice, show that SIRT1 has a role in preventing AD by promoting the non-amyloidogenic pathway of APP processing, a process associated with decreased levels of ROCK1 [46]. In our experimental model, overexpression of SIRT1, as well as their pharmacological activation also increases dendrite integrity and cell viability on hippocampal neurons exposed to Aβ aggregates, which therefore involves a mechanism independent of APP processing. NAD^+^ can supply the possible deficiency of cellular level of NAD^+^ present in neurons close to amyloid aggregate and activate SIRT1 pathway [47]. On the other hand, RES can act like a SIRT1 activator and regulates some transcription factors like PPARγ that protects against neurotoxicity of Aβ aggregates by preventing an increase of cytoplasmic calcium [48]. Another possibility involves forkhead proteins FKHR (FOXO1) and FKHR-L1 (FOXO3a) that prevent oxidative damage produced by Aβ aggregates, by a pathway that involves antioxidant proteins like Mn-superoxide dismutase and catalase [49].

In summary, we have demonstrated that SIRT1 and its deacetylase activity plays multiple roles in the development and maintenance of neuronal morphology by affecting dendritic branching and regulating dendritic spine morphogenesis. Potential targets of the desacetylase activity of SIRT1 could include the IGF-1 pathway. It has been reported that SIRT1 deacetylates in vivo and in vitro IRS-1 (insulin receptor substrate-1), a cytoplasmatic protein phosphorylated by the IGF-1 receptor [50], [51]. The interaction of IRS-1 with downstream effectors such as PI3K may inhibit RhoA/ROCK [52], [53]. Besides, it was shown in a mice model of AD that concomitantly with the increase in SIRT1 activity by caloric restriction, there was a decrease in ROCK1 protein levels, which may be associated to the effect of SIRT1 deacetylase activity over transcription factors such as FOXO and p53 [46]. Further studies will help to fully elucidate the mechanisms by which SIRT1 and its desacetylase activity influences neuronal morphogenesis and the formation of neural circuits.
